# Modified TIG Welding Joint Process: An Approach to Improve Microstructure and Fracto-Mechanical Behavior by MWCNTs Inducement in Al-Mg-Si Alloy

**DOI:** 10.3390/ma12091441

**Published:** 2019-05-03

**Authors:** Muhammad Muzamil, Jianjun Wu, Maaz Akhtar, Zengkun Zhang, Arfan Majeed, Junzhou Yang

**Affiliations:** 1 School of Mechanical Engineering, Northwestern Polytechnical University, Xi′an 710072, China; muzamil@mail.nwpu.edu.cn (M.M.); zhzk1987@163.com (Z.Z.); amajeed@mail.nwpu.edu.cn (A.M.); yjz@mail.nwpu.edu.cn (J.Y.); 2 Mechanical Engineering Department, NED University of Engineering & Technology, Karachi 75270, Pakistan; maaz@neduet.edu.pk

**Keywords:** TIG welding, MWCNTs, Pictorial model, microstructure, equiaxed grains, fracture behavior, pull-out fracture

## Abstract

This work provides a comprehensive investigation of multi-walled carbon nanotubes (MWCNTs) inducement in weldment and their apparent effect on the microstructure, %elongation and ultimate fracture behavior of Al-Mg-Si alloy referring modified tungsten inert gas (TIG) welding joints. Serious experimental work is carried out at 1 wt%, 1.5 wt%, and 2 wt% of MWCNTs to provide a gradually increasing heterogeneous nucleation. The behavior of grain morphology showed the pure field of epitaxial growth without MWCNTs, and the forestry type morphology for 1 wt% MWCNTs at low welding currents (160 A), though there was a noticeable conversion into equiaxed (EQZ) grains filled with inter-dendritic particles at high welding currents (180 A and 200 A) for 1.5 wt% and 2 wt% of MWCNTs. Moreover, the formation of a cellular type network above the fusion line predominated initially at all parameters. Conversely, fine EQZ grains were formed as they moved upward into the welded zone (WZ) explicitly at a high heat input. A conceptual pictorial model is presented in the study which summarized the behavior of morphological changes at the utilized parameters. The welded joints have demonstrated an increasing trend of strength and %elongation in contrast to joints without added MWCNTs. Comparative results have shown an exceptional increment of 71 to 76% and 67 to 75% of elongation up to ultimate tensile strength (UTS), and a fracture point that was clinched for 1 wt% and 1.5 wt% MWCNTs at 180A. From macro to micro-examination of the fracture surfaces, pure ductile modes constituting elliptical cup and cone type isotropic flow was evident in all specimens. Detailed confirmation of the pull-out fracture mode of MWCNTs has highlighted in the scanning electron microscope (SEM) images that intimated a methodical contribution in load-transfer from matrix to the fiber under axial load. Overall, a concise en-route for MWCNTs inducement is well-appointed through tube fillers along with an activating facilitator (TiO_2_) in contrast to stereotype fillers for improved behavior termed as modified TIG welding joint process in study.

## 1. Introduction

The curtailment of mass from elephantine structures in engineering applications is considered a decisive concern for automotive, aircraft, high-speed trains, and power utilities giant manufacturing industries [1]. Lightweight aluminum and magnesium alloys are considered promising structural materials and have been used extensively during the last decade [2]. In order to meet the requirement of acceptable strength, good formability, corrosion resistance, and welding ability, aluminum alloys are the prevailing excerpt. However, when the welding of aluminum alloys comes into forethought, tungsten inert gas (TIG) and metal inert gas (MIG) are the prime choices in the category of fusion arc welding processes because of their highly utilized industrial interaction along with high efficiency, quality joints, low cost, and excellent flexibility [3,4].

Despite the advancements in welding processes and an embryonic technology, several problems still exist and are critically identified as large heat affected zone (HAZ) formation, solidification cracking, coarse microstructure formation, defects including pores and voids, and softening phenomenon in heat-treatable alloys due to the thermal cycle effect [5]. Heat treatable aluminum alloys specifically experienced the worst deterioration in mechanical properties because of the impossibility of preventing the formation of softening zones in the weldment due to the nature of the fusion process. In the typical conditions of fusion welding, the weld zone exhibits coarse and long columnar grains subsequently upon solidification in the direction of the heating source, which is often termed the axial grain structure. It is highly recommended to develop an augmented mechanism to model the desired solidified structure in weldment but it is extremely difficult in the ubiquity of a high-temperature gradient for melting where epitaxial growth usually occurs in the axial direction from the chilled wall of the base material (BM). In order to break the consistency of epitaxial/axial growth at earlier stages, this study is drafted as a comprehensive novel solution of the above-stated problem.

It is espied in the most recent and popular trends in studies on Al-Mg-Si alloys that the fusion welding process enforced ER4043 and ER5356 filler. It is important to correlate that ER4043 contains high silicon (4.5–4.8%) to increase the fluidity, and ER5356 has almost 4.5% Mg content to provide more adequate strength to weldment. Both categories, however, belong to non-heat treatable aluminum alloys. Yan et al. have studied the comparative repercussion of ER4043 and ER5356 fillers on Al-Mg-Si alloy engaging the laser hybrid welding [6,7]. Estimations on mechanical performances have reported a reduction of micro-hardness and ultimate tensile strength (UTS) in comparison to BM. Proportionate read-ups on TIG and MIG welding have also reported a similar trend of reduction in yield strength, UTS, and micro-hardness against the developed heat input [8,9,10]. The accusatory reasons include the formation of different zones within a single regime, formerly called HAZ(I) and HAZ(II), which have experienced solution treatment and over-aging sequentially [11]. In order to circumvent this admissible behavior in different industrial applications, a distinct variant of available techniques is introduced in recent trends. Promptly, cold metal transfer (CMT), which is a combination of TIG and MIG, utilizing low heat input is currently a new research trend in welding. Liang et al. have presented a marvelous comprehensive investigation employing TIG-CMT hybrid welding by varying different parameters to provide a conclusive handout on microstructural and mechanical behavior using ER4043 filler. Depicting similar gravitate, 50% decrement in micro-hardness and strength along with a 40% reduction in elongation is reported in contrast to BM [12,13].

Broader aspects from the earlier discussion steer us towards high-cost advanced manufacturing systems that do not resolve the basic problem of heat treatable aluminum alloys welding. However, the other benefits include high productivity, improved surface finish, and thick section plate joining. In these circumstances, the flipside of the window should be tested which may control the microstructure and tailor the morphology of grain growth in accession with recent trends. Numerous researchers are galvanized to resolve the problem through the melding of nano inducements to control the microstructure behavior that ultimately affects the mechanical properties. Multi-walled carbon nanotubes (MWCNTs), which have already been declared as a next-generation material for structural application [14], are currently being used as a super reinforcement material for aluminum and magnesium alloys joints. Due to their low density, supporting interfacial reaction, multi-wall physical appearance along with excellent thermal stability, MWCNTs are in the limelight for outlining the augmentation for improvement in weldment competency. Apart from contributing in the strength, MWCNTs are also effectively used for grain morphology acclimation, normally varied in-between 1–3 weight percentages [15]. Khodabakhshi et al. introduced 2.5 vol.% of MWCNTs into friction stir welding as an effective grain refiner which provides nucleating sites in accelerating the effect of refinement [16,17]. Moreover, Fattahi et al. concluded that the formation of fine EQZ grains and conversion of dendritic structure (DS) equiaxed with increasing amounts of graphene nanosheets and MWCNTs through their various studies [18,19]. Due to the high aspect ratio of MWCNTs, a downward carrying mediator is obligatory to create a deep penetration effect without increasing the opening width of the weldment. However, this concern becomes pronounced due to the stamped nature of low weld depth penetration under the action of different forces acting on the weld pool, which include Marangoni flow, electromagnetic force, drag force of arc, and buoyancy force [20]. In that case, the increasing heat results in negative surface tension, which easily transfers the molten metal towards the edges by forming wide and shallow weldments. This dynamic contradicts the effective transferring of MWCNTs to the bottom of the pool, fortunately, the existence of oxide flux in integration changes the locomotion favorably. The surface tension turns positive by forming the shape of peanut-shell-like [21] weld beads due to the reverse flow of fluid. This guaranteed assistance is drafted from TiO_2_ which is reasonably famous for activating flux capabilities in conjunction with TIG welding [22,23]. The ability to transfer the fluid flow from radially outward to an inward central position due to the reversal of Marangoni convection [24] works exquisitely in the subjected case.

So far now, a microstructure-based comparison in the weldment showing how Al-Mg-Si alloys have been included in MWCNTs as nano inducement has not been presented dedicatedly and thoroughly. Here, the work sheds light on the effect of the MWCNTs coated AA6061 filler tubes as an application to break the consistency of epitaxial or axial growth of grains. A constant 1wt% of TiO_2_ is added as an activating facilitator for MWCNTs, which is an unmatched strategy in a group with former cases. This autocratic approach eradicates the axial cellular networks of grain at an earlier stage with explicit conversion into EQZ gains. Apart from microstructural improvement, %elongation and fracture behavior drive a novel-modification in the joint performance using inexpensive TIG welding process and obstruction to propose an expensive hybrid system. All the referred studies in the literature on microstructure and mechanical behavior utilized ER4043 or ER5356 fillers undone to represent the behavior of AA6061 as a filler. In addition, the utmost interest of the study is to portray a conceptual pictorial model through microstructure transformation from axial/columnar morphology to EQZ grains by providing heterogeneous nucleation. The overall impact shows that utilizing AA6061 as a nano-treated filler with MWCNTs is considered a distinct approach and will develop as a dominant second-generation filler for microstructure controlling. This study has compassed to be a dexterous blueprint to establish an explicit correlation on microstructure transformation and fracto-mechanical behavior through notable modification in existing manufacturing technique.

## 2. Materials and Experimental Procedure

### 2.1. Sample Preparation

**Figure 1:**
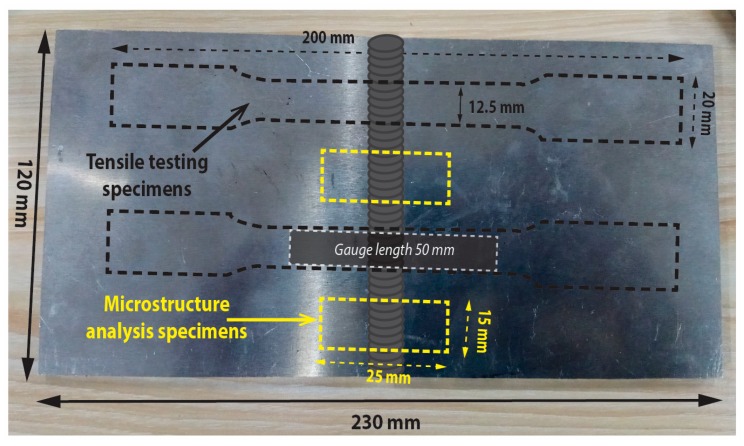
Pictorial illustration for the geometry of tensile and microscopic specimen extraction from Al-Mg-Si plates.

The welding base material used in the present research is Al-Mg-Si aluminum alloy provided in a plate of 5 mm thickness in the T6 condition. The rolled plates were sheared into the dimension of 230 mm in length and 120 mm in width in the direction of rolling. A V-type groove up to half of the thickness in depth was machined in the center of the plate for depositing filler. A comprehensive graphical illustration describing the extraction location of specimens for microstructure analysis and tensile are depicted in Figure 1. The 3 mm diameter hollow rods (AA6061) were used as the initial raw material with slightly greater Si contents to improve the fluidity of filler. The chemical compositions of both the materials were ascertained by a spectrometer (SPECTROMAXx) (SPECTRO, Kleve, Germany) given in Table 1.

### 2.2. MWCNTs Filler Fabrication

**Figure 2:**
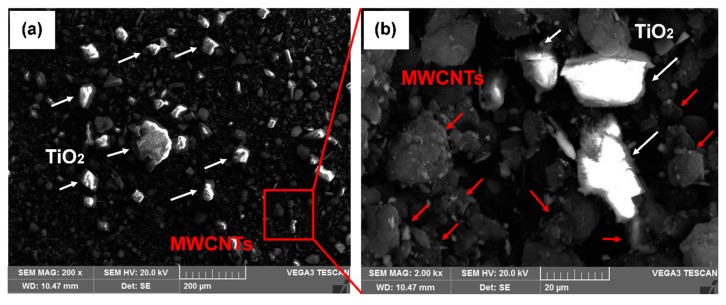
SEM micrographs (**a**) Ball-milled mixture representing multi-walled carbon nanotubes (MWCNTs) and disperse TiO_2_ particles, (**b**) magnified image of MWCNTs at a localized region.

In order to develop the fillers, Al-Mg-Si alloy tubes were tabbed and precisely sliced from the middle position using electric discharge machining to create a uniform opening. The process of filler fabrication was performed in two steps. The first step involved the utilization of MWCNTs (diameter 30 nm, length 20 µm) and TiO_2_ (40–50 nm) as the precursor materials for the production of homogenized suspension [25]. Starting with MWCNTs and TiO_2_ powder that were milled at low speed (250 RPM) to break-down the agglomeration and for achieving the uniform dispersion. The SEM images of the MWCNTs-TiO_2_ mixture are represented in Figure 2. The uniformly dispersed TiO_2_ particles are highlighted in Figure 2a, while the localized region for MWCNTs is magnified in Figure 2b. 

**Figure 3:**
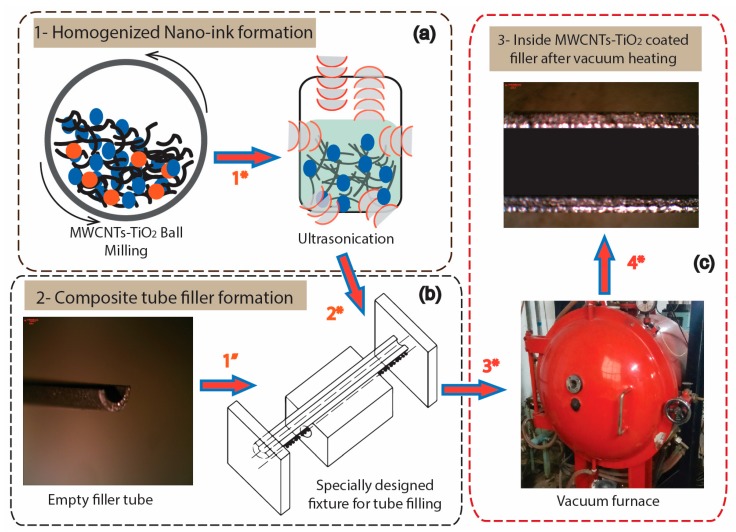
Pictorial illustration for the fabrication procedure of filler with foreign inducement in Al-Mg-Si tube; (**a**) homogenized ink formation, (**b**) tube filling with ink, (**c**) vacuum heating.

The obtained mixture was then ultrasonicated in the calculated volume of ethanol for 1.5 hours. The second step involved the filling of the desired volume of nano-suspension (ink) into the tubes, utilizing an in-house specifically developed fixture. All the tubes were properly scratched with a wire brush to remove the oxide layer and cleaned with ethanol prior to filling. The same sequence was followed for all the weight percentages of MWCNTs contained in the filler fabrication. The fillers were then further treated in a vacuum furnace at a constant cycle of 175 °C temperature for a holding time of 1 hour to improve the interface bonding between surface and nano-materials. A pictorial representation of stepwise fabrication of composite tube-filler coated with MWCNTs-TiO_2_ is represented in Figure 3.

### 2.3. TIG Welding Process

**Figure 4:**
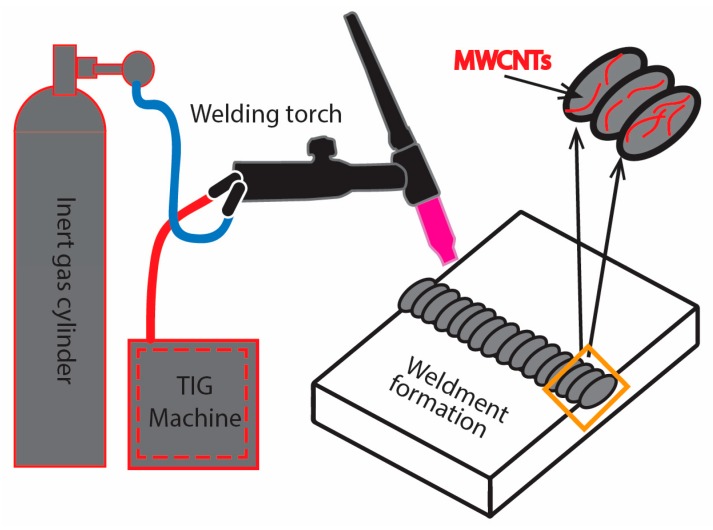
Pictorial illustration for the formation of MWCNTs induced weldment by tungsten inert gas (TIG) welding.

TIG welding was performed carrying a manually feeding torch gripping self-fabricated fillers as discussed in Section 2.2. The YC-500WX TIG welding machine (Panasonic, Osaka, Japan) operated on an alternating current was engaged to perform all the experiments. Pure argon gas with a flow rate of 18 L/min used as a shielding gas along with 3.2 mm tungsten electrode in the torch. Single-pass weld metals were made for the formation of the weldment at 160 A, 180 A, and 200 A along with 1 wt%, 1.5 wt%, and 2 wt% of MWCNTs contents. The experimental scheme was tracked by utilizing 1 wt% MWCNTs filler for weldments formation at 160 A, 180 A and 200 A, sequentially, which resulted in three specimens in particular. The same sequential order was followed for 1.5 wt% and 2 wt% of MWCNTs fillers, which resulted in six more specimens. Overall nine specimens were prepared, each repeated twice against the above set-in combination of current and MWCNTs-contents to validate the recurrence of results. A pictorial representation of weldment formation through TIG welding process is given in Figure 4.

### 2.4. Utilized Techniques for Characterization

The tensile strength evaluation of MWCNTs induced TIG welded specimens, as scheme written in Section 2.3, were assessed on CSS-44100 universal tensile testing machine (China Mechanical Testing Equipment Co., Ltd., Changchun, China). All the specimens were extracted from the weld plate using the standard guidelines of ASTM-E8/8 M-11 and tested at 2 mm/min of cross-head speed. The extracted microstructure specimens as highlighted in Figure 1 have gone through the standard adoption of grinding process at 400, 800, 1000, 1500 and 2000 SiC emery papers serially. For obtaining the mirror scratch-free surface, the diamond paste of 5, 2.5, 1 and 0.5 µm was applied while polishing. For microstructural examination, GX-71 optical microscope (Olympus, Tokyo, Japan) was utilized on etched specimens with freshly prepared Keller’s reagent using standard practices as written in ASTM-E407-07. The fracture surface analysis was conducted on SEM “Tescan Vega 3” (Tescan a.s., Brno, Czech Republic) equipped with energy-dispersive spectroscopy.

## 3. Results and Discussion

### 3.1. Microstructure Analysis and Pictorial Model

Microstructural based analysis in the weldment formation at a range of MWCNTs contents and welding current and through TIG welding has been investigated as a comparison of HAZ and WZ morphology. The formation of EQZ grains from columnar in typical solidified weld structure in practice is unlikely because of only a midget source being available in dendrites fragmentation and ceramic particles in filler as a nucleating agent. The purpose of adding hard particle (include Ti, TiB_2_, and Zr) in TIG welding commercial fillers is in compliance with the above-stated objective. It is considering possible with adequate heat input to create exceptional conditional for EQZ grain growth by providing nucleating sites through long aspect ratio MWCNTs. A limited number of works have exemplified the effect of cooling rate on grain morphology, and it is considered more complex attempt to compare what features are pop-upped by the varying current at 160 A, 180 A, and 200 A along with 1 wt%, 1.5 wt% and 2 wt% of heterogeneous nucleates on weldment morphologies.

**Figure 5:**
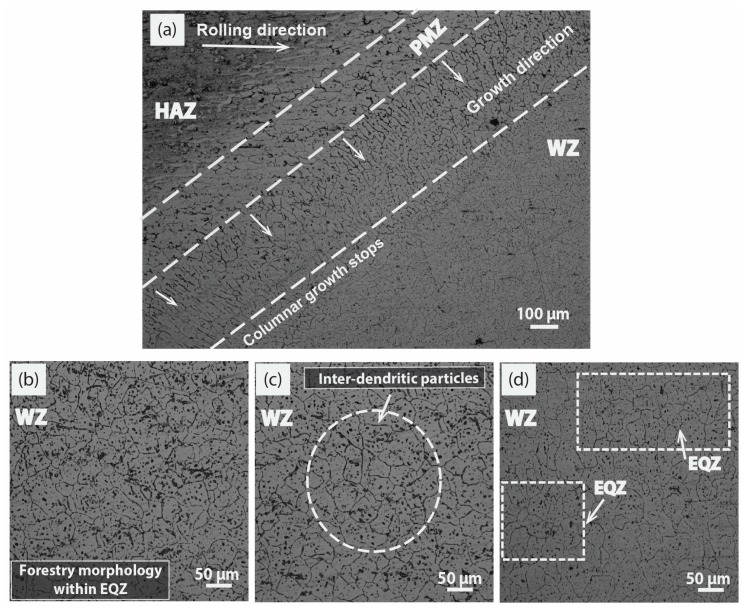
Optical microscope images at 1 wt% MWCNTs; (**a**) overview of weld at 160 A representing heat affected zone (HAZ), partially melted zone (PMZ), welded zone (WZ) (**b**) magnified WZ overview at 160 A, (**c**) magnified WZ overview at 180 A, (**d**) magnified WZ overview at 200 A.

**Figure 6:**
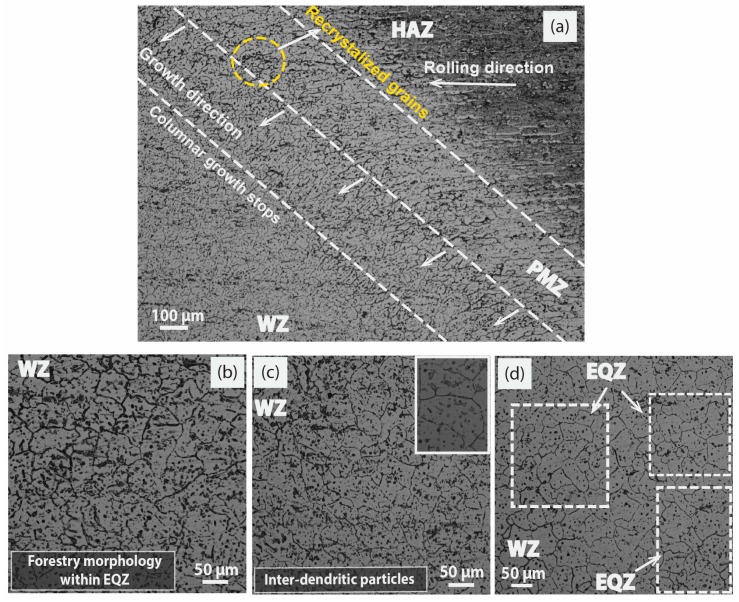
Optical microscope images at 1.5 wt% MWCNTs; (**a**) overview of weld at 180 A representing HAZ, PMZ, WZ (**b**) magnified WZ overview at 160 A, (**c**) magnified WZ overview at 180 A, (**d**) magnified WZ overview at 200 A.

**Figure 7:**
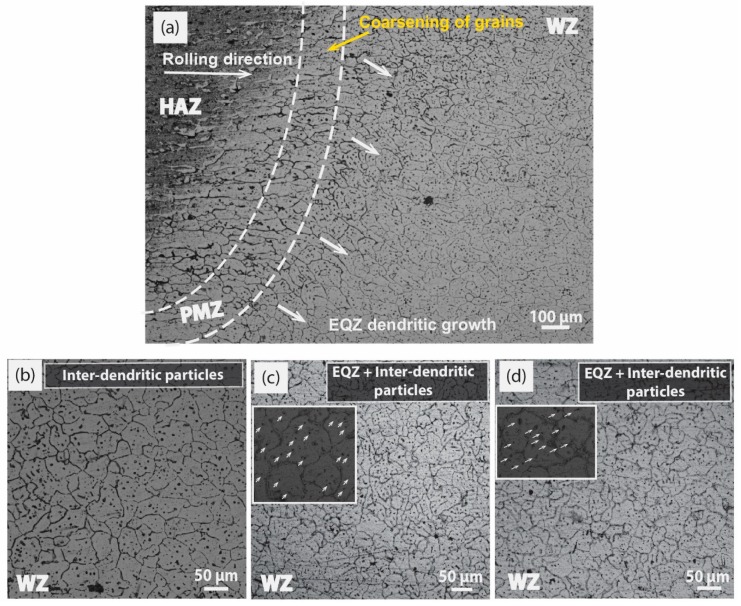
Optical microscope images at 2 wt% MWCNTs; (**a**) overview of weld at 200 A representing HAZ, PMZ, WZ (**b**) magnified WZ overview at 160 A, (**c**) magnified WZ overview at 180 A, (**d**) magnified WZ overview at 200 A.

All the observed micrographs are displayed in Figure 5, Figure 6 and Figure 7 as a function of the welding current and MWCNTs contents. It is quite familiar that the microstructure of the joint supports the formation of the long columnar structure either at high or low welding currents due to epitaxial behavior. The overview of the weld at 160 A and 1 wt% MWCNTs is depicted in Figure 5a, a distinct fusion line is separating the HAZ, partially melted zone (PMZ), and WZ, and a columnar axial growth is evident which travels perpendicular from fusion line towards the WZ as indicated with arrows. However, the HAZ exhibits an inhomogeneous coarse cellular network of grains in the direction of rolling.

It is to be noted in Figure 5b, the effect of the long columnar completely diminished as WZ advanced and converted into irregular forestry dendrites within EQZ grains due to the addition of 1 wt% MWCNTs. Moving to step ahead in current to 180 A in Figure 5c, the behavior of the WZ turns into a clearer EQZ structure [26,27] and the evidence of inter-dendrites within grains in the form of particles are available at many instances, highlighted in the micrograph. Though the overview of the weld section shows a slight change in the morphology just above the fusion line; a representation of the stray type intermediate columnar structure due to relatively high heat input. With a further increase in current to 200 A, the overview of the WZ is represented in Figure 5d. The slightly coarser EQZ grains were formed in the central region along with fine dendritic particles in the micrograph. Like in the previous case, inter-dendritic structure exists within a grain but this time particles like dispersion are less dominant due to high heat input than Figure 5c. A distinct change in morphology was concluded at the 200 A current at a constant rate of nucleation inducement (1 wt% MWCNTs) in contrast to 160 A and 180 A. In the WZ, the coarse EQZ grains were a witness at 200 A, however, a noticeable decrement in the grain boundary (GB) density is an extraordinary feature shown by increasing the welding current.

Typically, an increase in current, which is an adequate parameter for adjusting and improving response, created coarser microstructures in the WZ, and more prominent PMZ and a larger HAZ that caused a substantial drop in the mechanical properties. A slight increase in nucleation percentage from 1 wt% to 1.5 wt% of MWCNTs was exercised to break the consistency of the coarse columnar grain structure into more EQZ grains. The conditions provided by Hunt [28] for the conversion of the columnar to EQZ required the continuous supply of nucleation for new growth of grains, which must match the growth rate of a weld pool in compliance with favorable thermal conditions. The formation of the weldment structure at 180 A by 1.5 wt% MWCNTs is displayed in Figure 6a at the overview of HAZ and WZ. The growth of columnar dendrites above the fusion line still continued in the direction of achieving maximum heat input and became eventually frozen at a certain length as highlighted (columnar growth stops) in the overview. Moreover, the overview has depicted a distinct broad fusion line formally called the PMZ. A mixture of recrystallized and dendritic grains was highlighted in the PMZ, following which, columnar solidification started in the direction of the heat source. A similar dendritic forestry morphology in the WZ as achieved in Figure 5b is repeated in Figure 6b with smaller grain sizes due to increased nucleation sites (1.5 wt% MWCNTs) at 160 A. 

The optimized amount of heterogeneous nucleate sites for EQZ grains may be explained well from the thermodynamic perspective, a further step can go ahead in heat input by increasing the current to 180 A at constant welding speed [29]. A kind of supercooling effect is created for 1.5 wt% MWCNTs at 180 A, as shown in Figure 6c. An increase in heat input that increases the cooling rate of weld metal and taking further advantage from conductive MWCNTs in the weld pool allows directional heat flow by convection to the top surface. As shown in Figure 6c, the inter-grain region of the WZ became filled with fine dispersoids particles with a high population intensity that was highlighted separately in the intensified box. The mechanism included the nucleation of new grains with fine dispersoids in the weld pool, which further blocked the growth of the long columnar structure [30]. Ultimately the formation of EQZ and fine inter-dendritic structure increased the GB density, which overall resulted in an improvement in mechanical properties. In the next upgraded level of welding current at 200 A, an increase in heat input value decreases the cooling rate at the constant welding speed. This results in the refined EQZ grain boundaries that were tagged in Figure 6d as gradually moving up in the WZ, but the density of inter-dendritic particles is exceptionally reduced in comparison to Figure 6b,c.

For providing more heterogeneous nucleation in the formation of enormous new grains and morphology refinement, 2 wt% MWCNTs were inserted inside the composite tube filler as explained in Section 2.2. It was observed that the temperature gradient (G) and growth rate (R) ratio G/R had solicited consideration on the final morphology of the grain, and a low G/R ratio was endorsed for fine grains and a constitutional supercooling effect. Inducement of 2 wt% MWCNTs at low heat input (160 A) had compatibility issues with the high nucleation rate due to the low wettability of MWCNTs with the matrix. Consequently, incomplete fusion and incipient melting of GB may arise. Somehow due to the high nucleating effect, an adequate level of fine grains was reported in the WZ as depicted in Figure 7b, but the effect is not so pronounced as to create inter-dendritic dispersoids within the grains as in previous cases. 

**Figure 8:**
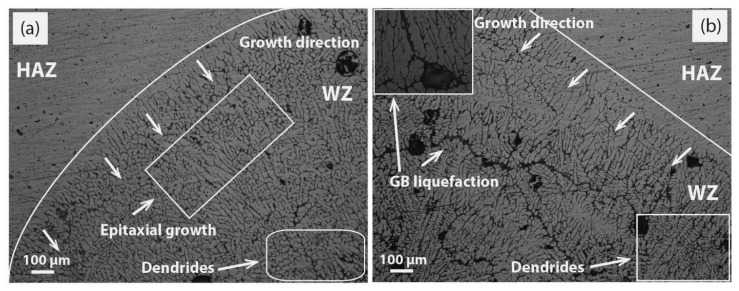
Optical microscope images of without MWCNTs weldments; (**a**) overview of weld at 160 A (**b**) overview of weld at 200 A.

It is reported in studies [29,31] that high heat input and welding speed form EQZ grains by the formation of heterogeneous nuclei in the weld pool. The only increment is welding speed, and this is not a feasible condition in engineering applications for welding large structures because other defects may occur, such as improper fusion and a low depth to width ratio. A more homogenized WZ is presented in Figure 7c, at a moderate heat input of 180 A with slightly high 2 wt% MWCNTs. This will create a more favorable condition in providing the external nuclei ahead of the solidified dendrite front, which was experimentally pictured in WZ with well-EQZ grains filled with fine dispersoids. Moreover, to provide high heat input in combination with high welding speed to give less time to the available hard particles for dissolution is not an appropriate task. It will take multiple cycles of heat to dissolve particles like Ti, as one case is reported in Figure 12f (Section 3.3) of other facilitated TiO_2_ particles as an activating flux. As planned in the experimental design, more heat input value is utilized at 200A to increase the fluidity of the weld pool and wettability with 2 wt% MWCNTs. More effective long aspect nuclei created the conditions in the favor of EQZ grains as represented in Figure 7a,d. An almost very limited PMZ was created at the interface of a weldment in Figure 7a and the formation of fine dendrites and EQZ grains were visible right from the start of the fusion line. Apart from creating EQZ dendritic grains in Figure 7d, fine dispersoids have also created optimized conditions at a localized region. Figure 8a,b has represented the weld microstructure overview at 160 A and 200 A without MWCNTs. As directed in Figure 8a, a continuous columnar and epitaxial growth is a witness over the entire field of the microstructure. However, in Figure 8b, more prolonged elongated growth is depicted due to more heat input induced at 200 A. In addition, a self-styled crawling stray morphology, which was relatively small in size and similar in shape to columnar, existed in the WZ for both cases. Though other characteristic features, including GB liquefaction, are evident in Figure 8b, it represents a strong network in connection with porosities. The purpose of the inclusion of microstructures in Figure 8 is to provide a concrete comparison between them with and without MWCNTs heterogeneous nucleation for consolidation on the pictorial model.

**Figure 9:**
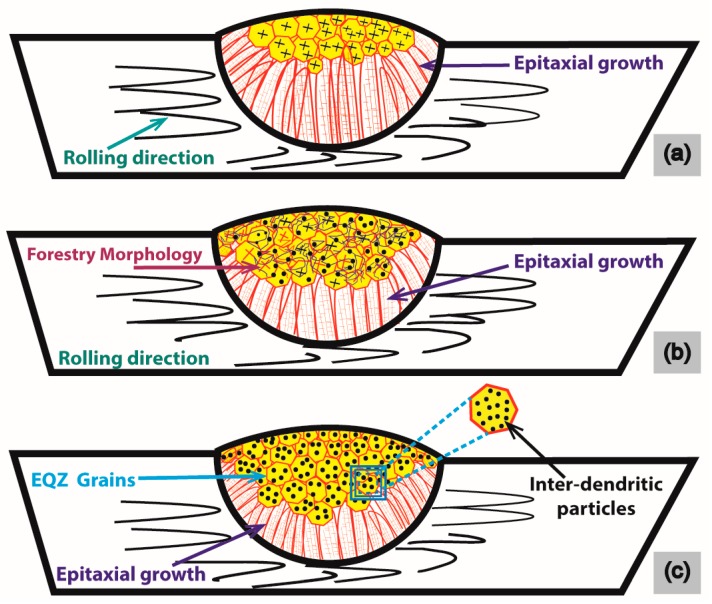
Pictorial model representing the behavior of microstructures; (**a**) pure epitaxial behavior, (**b**) forestry morphology, (**c**) inter-dendritic dispersoids morphology in EQZ grains.

Finally, a pictorial model was extracted from the behaviors of microstructures representing the stage-wise conversion of columnar morphology through epitaxial growth into complete EQZ grains by the addition of MWCNTs. The experimental morphological conclusion in the form of the model is outlined in Figure 9. For a simplified illustration of Figure 9a, the straight unremitted columnar grains are formed from the BM wall, which acts as the initial source of nucleation, and the solidified structure continuously grows above the fusion line formerly called epitaxial growth. The likelihood of EQZ crystals is quite low, however, the solidified structure usually forms columnar dendritic texture [32] as perfectly depicted in Figure 8 for the micrographs without MWCNTs. Moreover, the high cooling rate could constitute EQZ dendrites at the limited top surface of the weldment. Apart from the change in microstructural morphology, the observation in the dynamics of GB density shows another variation that has occurred with the change in heat input current and MWCNTs contents. As a normal trend, GB density decreases by increasing the welding current due to the high temperature experienced for a long time, which favors this condition. For the cases discussed in Figure 5b and Figure 6b, the inter-dendritic particles expanded like irregular branches of a tree (forestry) within the grains. However, for rest of the cases are a combination of branches and more orderly decorated nuts within a grain. Initially, at a low welding current of 160 A and 180 A, the thermodynamics conditions favor the formation of columnar type structure, though converted to forestry (irregular branches of trees) type dendritic structure or particles due to continuously providing (1 wt% MWCNTs) nucleation as pictured in Figure 9b. For the second case, thermal conditions oriented into a high solidification rate at high heat input current with a high nucleation rate (1.5 wt% MWCNTs & 2 wt% MWCNTs) as directed in Figure 9c. Gradually the grain morphology turned towards forming EQZ grains with inter-dendritic dispersoids filling. A relative compensation in GB density is referenced due to the formation of inter-dendritic dispersoids as discussed earlier in contrast to forestry morphology.

### 3.2. Strength and %Elongation Relationship with Current and MWCNTs Contents 

**Figure 10:**
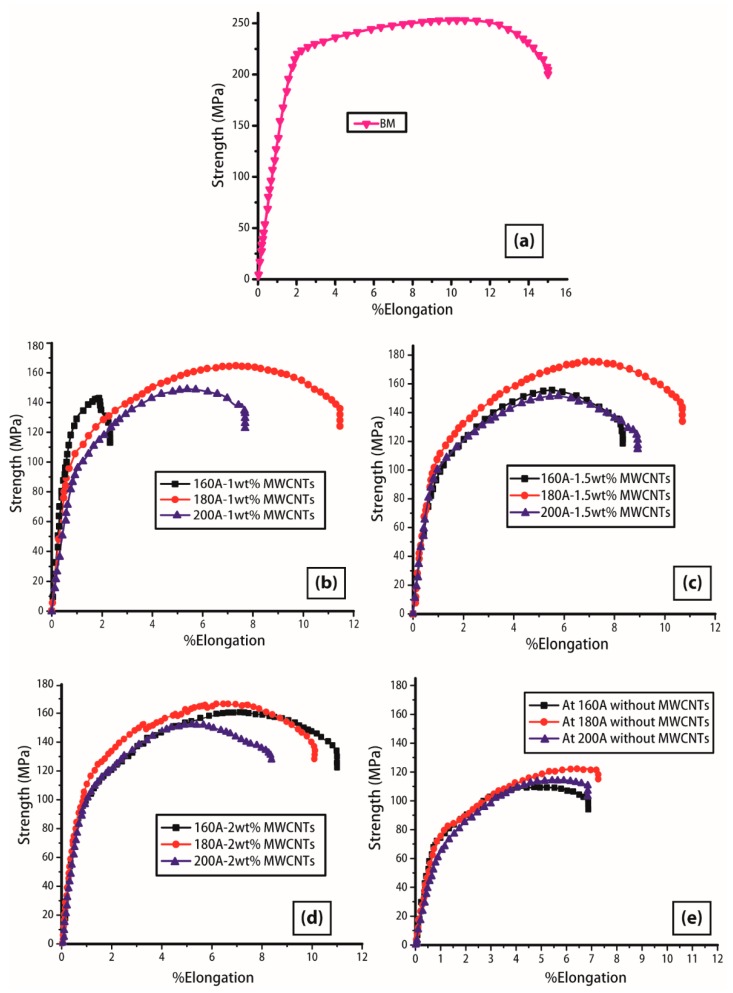
Strength and %Elongation plot; (**a**) Base material (BM), (**b**) 1 wt% MWCNTs, (**c**) 1.5 wt% MWCNTs, (**d**) 2 wt% MWCNTs, (**e**) without MWCNTs.

The value of ultimate tensile strength usually increases with the increase in welding current starting from low values (100–150 A) [33], though the behavior is hard to find a trend above 150 A. The addition of different weight percentages of MWCNTs is an additional radical on conclusive verdicts of results. All the results of tensile tests are presented in Figure 10 in the form of graph plotted between strength and percentage elongation. The graphs are divided into different blocks by varying welding current (160 A, 180 A, and 200 A) and MWCNTs kept constant for one experimental set as interpreted in Section 3.1, to show their aiding relationship with the microstructures. The inducement of MWCNTs is in under developing stages for the TIG welding process, hence, to conclude, the uniform and exact behavior on strength and elongation is quite difficult due to the variation of properties within welding technologies.

As interpreted in Figure 10b, an increasing trend in strength is depicted with an increase in current from 160 A to 180 A relatively with a slight reduction at 200 A using 1 wt% of MWCNTs. The first specimen at 160 A was fractured from the joint position with the least UTS, however, the investigation of failure which is well-explained in subsequent Section 3.3. The highest UTS of the block was achieved at 180 A due to EQZ grain structure formation in WZ that was filled with inter-dendritic particles as evidenced in Figure 5c. However, a reduction in strength was reported due to high heat input at 200 A that caused the dissolution of particles in Figure 5d. It is obvious from previous and recent studies that experiencing a reduction in UTS in contrast to BM properties from the adoption of conventional or any variant of the TIG welding process is reasonably normal. It can be seen from Figure 10c that an improvement in strength comes from figuring out how to use 1.5 wt% MWCNTs under similar welding current levels, in contrast to Figure 10b. The maximum UTS of the block (175 MPa) is reported at 180 A and 1.5 wt% MWCNTs, due to providing a supercooling effect to the weld pool, and in parallel limiting the dissolution of Mg_2_Si precipitates in HAZ. An intermittent effect of strength is captured in Figure 10d at 2 wt% MWCNTs which endorsed the performance slightly lower than 1.5 wt% (Figure 10c) but well-improved results from the 1 wt% addition of MWCNTs in Figure 10b. The purpose of modification in TIG welding is not to increase the properties but to limit the loss during fusion and their impact on adjacent zones. It is important to notice and highlight the effect of work hardening and work softening in all the curves up to the final fracture as presented in Figure 10 with the BM strength-elongation curve. The response of curves required additional stress (MPa) beyond the yield strain to produce further deformation (word hardening) [34] and this achieved plastic deformation was continually resisted by nor increase or decrease in strength (work softening) [35] as for BM in Figure 10a. For better understanding, the obtained strength-elongation curves are plotted in Figure 10e at 160 A, 180 A, and 200 A for without MWCNTs. The above-discussed phenomenon is clearly identified for cases at parameters; 180 A–1 wt% MWCNTs; 180 A–1.5 wt% MWCNTs, 160 A–2 wt% MWCNTs, and 180 A–2 wt% MWCNTs as likely in BM, anyhow lacking was observed in cases for without MWCNTs.

**Figure 11:**
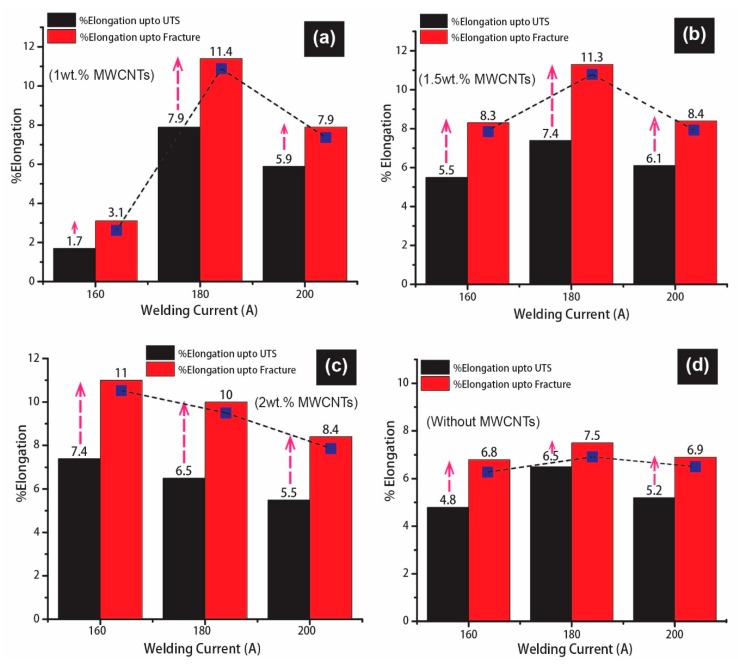
%Elongation vs. welding current relationship; (**a**) 1 wt% MWCNTs, (**b**) 1.5 wt% MWCNTs, (**c**) 2 wt% MWCNTs, (**d**) without MWCNTs.

It is equally important for the same concern to summarize the effect of similar parameters on %elongation up to UTS and fracture point. All the plotted bar graphs are represented in Figure 11 in a systematic comparison manner as both the elongations are critically important for engineering applications. The highest values of elongations were reported for BM that was 11% and 15% at UTS and ultimate fracture, respectively. The ductility of bulk materials is under the inverse relationship with strength, but it has become complex, and dependency on experimental results is more pronounced where different exhibited zones of mechanical properties come in tension, namely WZ, PMZ, and HAZ. Figure 11a shows the graphs between %elongation and the welding current representing the ultimate elongation of the system (180 A and 1 wt% MWCNTs) i.e., 7.9% and 11.4% at the UTS and fracture, which is 71% and 76% of BM. Afterward, a declining trend is reported at 200 A due to high heat input causing the deterioration of properties.

An increasing trend can be observed from Figure 11b from 160 A to 180 A with 1.5 wt% MWCNTs and a similar declining effect at 200 A, as in the previous case. The maximum %elongation of 7.4% and 11.3% are reported at 180 A, which is 67% and 75% of the BM. However, a well-balanced and stabilized trend is depicted in Figure 11b in contrast to Figure 11a. A relatively slight decline in %elongation is depicted with the increase in current at 2 wt% MWCNTs in Figure 11c within the same regime, though this case displayed more stabilized and improved results than Figure 11a,b. More importantly, a significant increase in %elongation from UTS to fracture is highlighted with arrows due to sufficient resistance provided by HAZ in plastic deformation. The results assure the blockage of dissolution of precipitates because of MWCNTs inducement.

**Figure 12:**
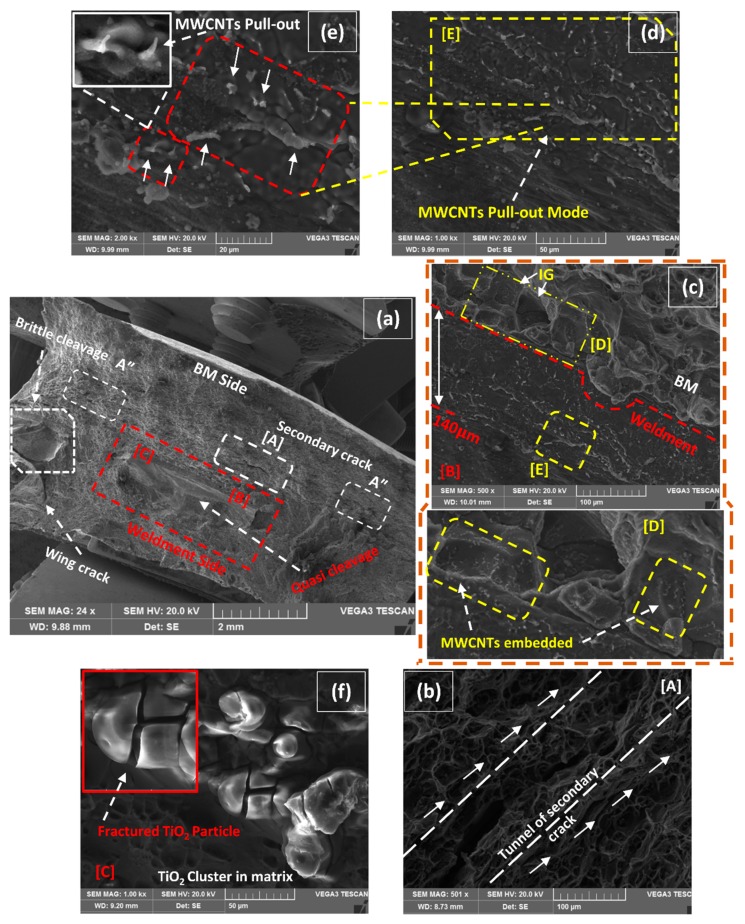
(**a**) Macro fractured image of 1 wt% MWCNTs–160A, (**b**) detail of [A] representing the tunnel of secondary crack, (**c**) detail of [B] representing the fractured interface of BM & weldment, (**d**) detail of [E] representing the pull-out mode of MWCNTs, (**e**) enlarged view of pull-out fractured MWCNTs, (**f**) presence of fractured TiO_2_ particles at location [C].

**Figure 13:**
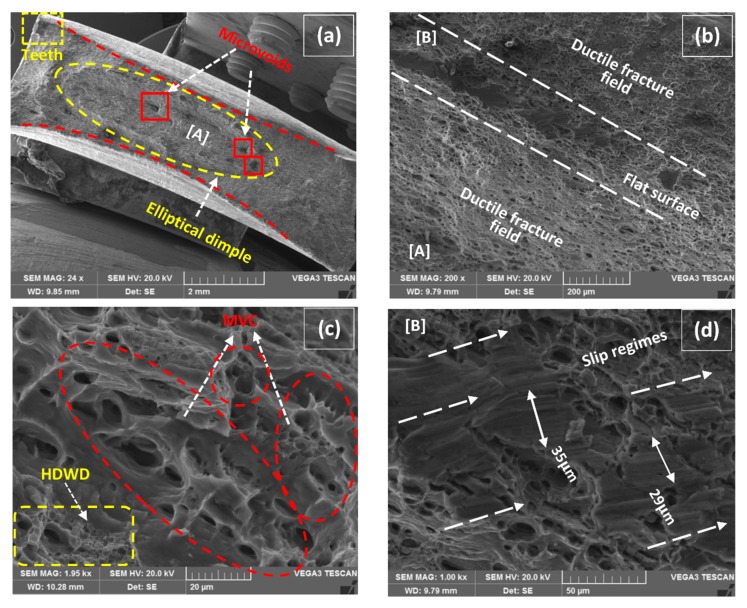
(**a**) Macro fractured image of 180 A–1 wt% MWCNTs, (**b**) detail of [A] representing the pure ductile field, (**c**) magnified image representing microvoid coalescence (MVC) and high density of weeny dimples (HDWD), (**d**) detail of [B] representing slip regimes.

**Figure 14:**
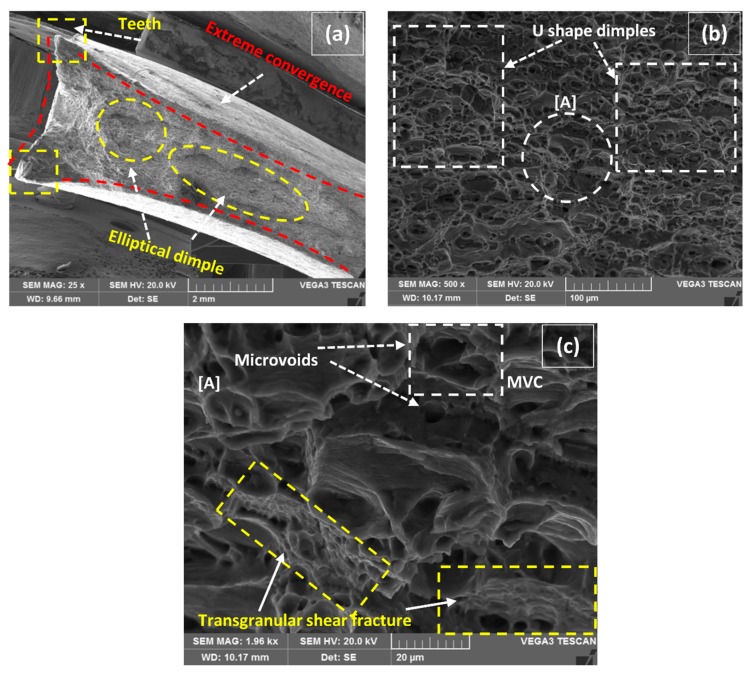
(**a**) Macro fractured image at 180 A–1.5 wt% MWCNTs, (**b**) magnified section representing U-shape stretched dimples, (**c**) detail of [A] representing MVC and transgranular shear fracture (TSF).

**Figure 15:**
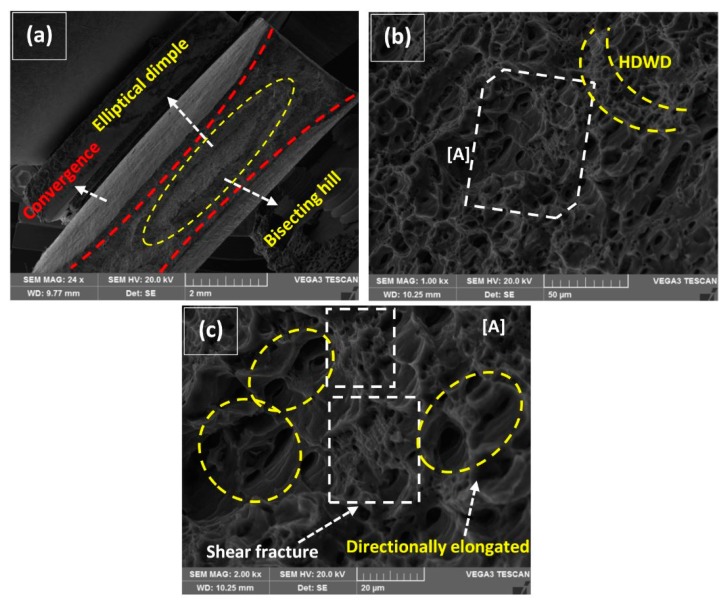
(**a**) Macro fractured image at 160 A–2 wt% MWCNTs, (**b**) magnified section representing directionally elongated dimples and HDWD, (**c**) detail of [A] representing packets of shear fracture.

**Figure 16:**
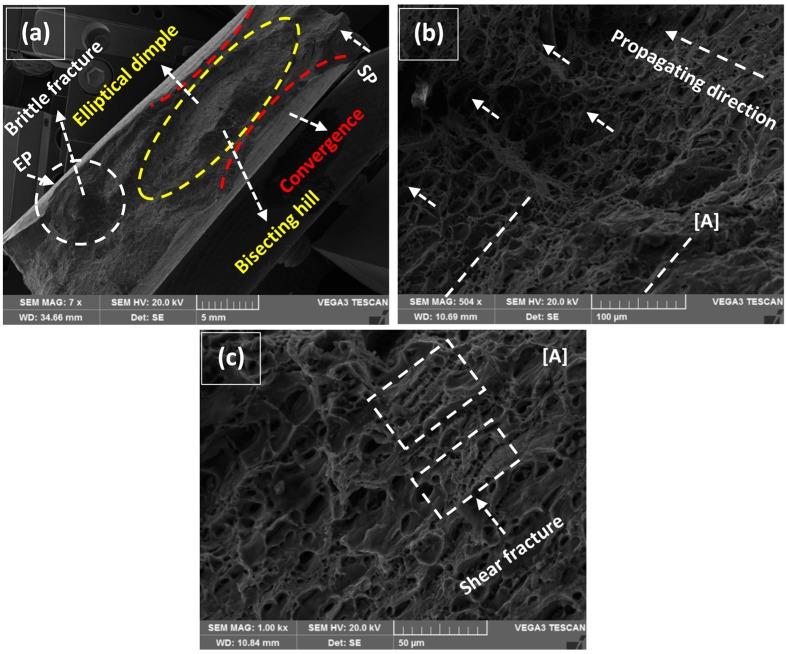
(**a**) Macro fractured image at 160 A-without MWCNTs, (**b**) magnified section representing directionally elongated dimples, (**c**) detail of [A] representing packets of shear fracture.

Due to enough furnishing of time to dissolve precipitates, along with increasing heat input at 160 A, 180 A and 200 A in Figure 11d, without MWCNTs, there is a reported gradual reduction in %elongation values. The trend that is revealed provides almost the least values in comparison to the results displayed in Figure 11a–c, except one case at 1 wt% MWCNTs–160 A. Excluding this, all the specimens were fractured within HAZ along with excessive necking before fracture, which is highlighted through SEM micrographs in Figure 12, Figure 13, Figure 14, Figure 15 and Figure 16. This suggests that weldments have higher strength due to the MWCNTs strengthening mechanism, and HAZ still possess the lowest plastic deformation ability. However, the locality of fracture is dependent upon the experience of heat that formed further two zones HAZ(I) (over-aged) and HAZ(II) (softening-zone) within HAZ. This mechanism of increase and decrease in elongation is possibly better interrelated with the age-hardening dynamics of HAZ (AA6061) from two concerns. Firstly, in the T6 condition, the foremost barriers for dislocation movement are the placement of β″ needle-shaped precipitates (Mg_2_Si) in a crowded population that provides higher strength as represented for BM in Figure 10a. Secondly, enough time during welding at elevated temperature in presence of MWCNTs resisted the heat directionally (divert to downward TiO_2_, restrict expanding to sideways) and partial reduction of barriers caused relatively high elongation percentages in Figure 11a–c. While for without MWCNTs (Figure 11d), complete reduction of barriers reduced the flow stresses though induce extra ductility similar for the cases in solution treatment.

### 3.3. Fracture Surface Analysis

The typical characteristics of tensile specimens fractured in Quasi-Static deformation conditions are represented in Figure 12, Figure 13, Figure 14, Figure 15 and Figure 16. All the fractured specimens were examined utilizing SEM, Tescan Vega 3, to conclude the macro and microscopic features in the form of a collectively logical mechanism. The strategy involved the presentation of macroscopic fracture surface that revealed the overall fracture mode at low magnification, subsequently proceeding with high magnification fracto-graphs provides the information in microscopic level at the acquisitive localized region.

Figure 12a has represented the macrographic fracture surface of the specimen that was fractured from the joint position (160 A–1 wt% MWCNTs). The ultimate cause of this failure is pickup through several clues that are highlighted in the same and explained in subsequent images. The two ultimate causes include; (1) quasi-cleavage at the bottom interface of the weldment, (2) secondary cracks (SC) growth in the transverse direction of axial force just adjacent to the weld seam line. As highlighted in Figure 12a, the locally radiating river lines moving towards the weak zone are the characteristic feature of quasi-cleavage [3]. The lines are sickly radiated and almost flattened in nature because it is not a crystallographic failure. Primary cracks are pre-existing (flaws) forward tensile cracks that trigger SC and propagate in a specific line of action [36,37], as marked with detail [A] in Figure 12a. Occasionally SC has also endorsed as shear crack, as pointed out the traveling from [A] to A″ at the fractured surface. The magnified detail of [A] is represented in Figure 12b, which represented a tunnel formed SC traveling forward (towards A″) varying from 15 µm to 300 µm in width. The above discussed intrinsic behaviors have prevented the formation of stable crack growth and led to eventual joint failure.

Moreover, the other important features at the interface of BM and weldment are marked with detail [B] and exemplified in Figure 12c. The upper side of the interface represents the fracture morphology of BM while the lower side belongs to the weldment. The first fractured layer in BM just above the interface has maintained intergranular (IG) facets up to a few grains due to the trapped sandwich in-between the quasi-cleavage and SC. Though a transformation has occurred from intergranular to transgranular, it has moved away from the interface. As marked with detail in [D] in Figure 12c, it is magnified as an extension representing the embedment of MWCNTs at the surface of the facet as highlighted. A graded rough surface of weldment below the interface (detail [E]) is augmented in Figure 12d, depicting the pull-out fracture mode of MWCNTs.

Though different strengthening mechanisms have been proposed and considered responsible for weldment; such as shear lag models, thermal mismatch, Orowan dislocation looping and grain boundary strengthening [18,38,39]. These mechanisms could be only effective when an acceptable bonding between matrix and MWCNTs existed. In the current scenario where the other influencing factors fail the weldment from the joint, the traces of MWCNTs as effective reinforcing agents still works functionally covering the significant area under pull-out mode as highlighted in Figure 12d. Figure 12e is taken at a high magnification of Figure 12d, representing the pinpoint locations of pull-out fracture mode of MWCNTs against the axial load. As the highlighted box in Figure 12e confirms, MWCNTs survived at that heat input and were well distributed inside the weldment. This further suggests that the load was transferred from the Al-matrix to MWCNTs in contributing to the shear stress component, as suggested in the model proposed by Kelly and Tyson [40]. Though many researchers give credit to the fractional formation of Al_4_C_3_ by a chemical reaction that locked the position of carbonaceous material in the matrix for contributing apart in an effective strengthening mechanism [41,42]. The other important aspect is the addition of TiO_2_ for the activate fluxing of molten in the WZ region that provides a deep penetration inducement to MWCNTs. The position marked with detail [C] in Figure 12a is delineated in Figure 12f which embellishes the clusters of TiO_2_ in the matrix at the extreme interface of BM and WZ. Moreover, the formation of the 140 μm band in Figure 12c of MWCNTs inducement up to the acute interface (depth) did not come out without the annexation of TiO_2_ for offering flux action in the weldment.

Figure 13a has represented a classical macrograph of highly ductile fracture attainment of the tensile specimen at 180 A with 1 wt% MWCNTs. The initiation of fracture has occurred from the center position by forming a large elliptical dimple in-between as highlighted in the figure. The shape of the fracture mode is exactly in resemblance with cup and cone type, though the cross section is rectangular (not circular) which grows with the same aspect ratio in longitudinal and transversely towards the periphery forming isotropic elliptical flow [43]. The presence of microvoids at distributed positions can easily be seen in Figure 13a. Moreover, the formation of plastic deformation before fracture caused the extreme convergence of the longitudinal side towards the transverse as marked separately forming baby teeth at the edge. A magnified image of detail [A] in Figure 13a is presented in Figure 13b describing the fields of pure ductile failure. The involved mechanism is the generation of numerous voids from the available cracks and particles, or secondly, the already available microvoids open up broadly by plastic deformation [44].

It reaches a relatively large spherical shape make coalescence with another void, formally called ductile fracture by microvoid coalescence (MVC) [45], as depicted in Figure 13c. An array of respective MVC is highlighted representing the enlargement of voids propagating towards the weakened zone of the structure. Another alluring detail is highlighted in the similar figure at the extreme bottom representing the high density of weeny dimples (HDWD) around the void concluding the overall ductile fracture. Moreover, a low energy flat surface (slip regime) [4] between two ductile fields is marked in Figure 13b with detail [B] enlighten us in Figure 13d. It is to be noted that every regime of slip originated after one another in a successive series of short in length (<45 µm) by forming separate forward flutes. In addition, slip regimes have a clear distinction with cleavages patterns that form high energy rivers lines originating from a single point relatively two or three times greater in length. A significant correlation exists between this pure ductile mode of fracture with Figure 10b for providing high values of elongations at UTS and up to fracture.

Figure 14a has represented the macro fracture surface of the tensile specimen that gave the maximum UTS at 180 A and 1.5 wt% of MWCNTs. The nucleation of the ductile fracture again alluded to the cup and cone mode by forming fragmented elliptical dimples at the center position, as in the previous case. Conjointly, an extreme level of convergence from sideways in HAZ is visible as a major constituent of ductility due to plastic deformation by forming high heel sharp teeth at the edges. A magnified section in Figure 14b represents the ductile enlargement of dimples in U-shape that were spread throughout the captured field [46]. The U-shape dimples seem to be extra stretched forming a relatively shallow and flattened nature of field representing shearing fracture. It is confirmed that HAZ still possesses enough secondary phase particles to provide the strength that shallow elongated dimples and microvoids formed ductile transgranular fracture [47] as the ultimate result. A pointed region with [A] in Figure 14b is further magnified in Figure 14c for more clarification of the fracture mode. A similar mechanism of MVC is followed in Figure 14c as earlier tracked in Figure 13c, the nucleation, growth, and coalescence of microvoids, which were categorically spotted, though the secondary phase particles were not visible at that magnification. It is imperative to notice the affirmation of transgranular shear fracture (TSF) that is highlighted in patches which represents a greater amount of plasticity associated with failure.

Comparative fracture macrographs at 160 A with 2 wt% and without MWCNTs are represented in Figure 15a and Figure 16a. A similar cup and cone forming elliptical dimple are characterized by the classical ductile fracture mode along with little change in macro features. The longitudinal length of an elliptical dimple in Figure 16a is significantly decreased in contrast to 2 wt% MWCNTs addition. In tallying, the sideways convergence towards transverse is relatively higher for 2 wt% than without MWCNTs that was considered as an ultimate reason for providing 11% (2 wt%) negating to 6.8% (without MWCNTs) elongation at fracture in Figure 11c,d. It is to distinguish that a bisecting hill is present which divides the elliptical dimple, though a smooth-mild hill is evident for 2 wt% against the steeper one for without MWCNTs. The track of fracture is much more prominent in Figure 16a, where the starting point (SP) enters in the vicinity of ductile mode traveled along the sideways of the hill and finally formed a circular brittle facet, which is the end point (EP) of rupture. Both the fractured micrographs in Figure 15b and Figure 16b represent the directionally elongated coarse and small dimples. Though the epidemic fracture appearance of both has represented deep dimples in contrast to the previous case represented in Figure 14b, a fuzzy and corrugated type surface is more tangible in Figure 16b. The details of the marked region [A] in Figure 15b are magnified in Figure 15c representing two tributaries of dimples. The first tribe includes the moderate density of coarse dimples and second interpolates the high density of localize small dimples coming out of the plane [48]. A similar trend of tribes is also experienced in Figure 16c, but with relatively flattened features.

More importantly, the attention was drawn from forsaken packets of shear fractures in Figure 15c and Figure 16c that are more helpful to provide a superlative work-hardening effect by the ligament type shear failure of dimples. These randomly distributed packets improved the micro-plasticity at the localized regions under axial loading conditions as the high behavior of UTS and elongation is already concluded in Figure 10 and Figure 11.

## 4. Conclusions

Supplemental attention has been given to MWCNTs, which introduce a significant improvement in microstructure and mechanical behavior and open up another scheme of restructuring. The sententious characteristics of grain morphology, overall strength, %elongation and fracture behavior due to modification in TIG welding joint process are summarized as follows:

1. A pictorial model-based behavior has been developed from the conclusive experimental results through MWCNTs inducement in the weldment. Three behaviors are evident in the model, which include pure epitaxial/axial growth, forestry morphology and inter-dendritic particles filled grains for without MWCNTs, 1 wt% MWCNTs at low heat input and 1.5 to 2 wt% MWCNTs at high levels of heat input, respectively.
2. With the increase in welding current considered an adequate task for adjusting properties, decrement in GB density is evident at 180 A and 200 A. A kind of supercooling effect was created at 1.5 and 2 wt% addition of MWCNTs to provide compensation in GB density by the formation of inter-dendritic dispersoids.
3. The behavior of %elongation at high input values is hard to find because the available results are valid for low values of welding current. This study has concluded the response at 160 A, 180 A, and 200 A of current on %elongation at UTS and up to ultimate fracture. The generalize trend has depicted an increasing behavior for both the elongations at 160 A and 180 A, although a slight declining effect is reported at 200 A. The ultimate values of 71 to 76% and 67 to 75% of elongation in contrast to BM at UTS and up to fracture points are reported for 180 A–1 wt% MWCNTs and 180 A–1.5 wt% MWCNTs, respectively.
4. A detailed confirmation for the nature of fracture in weldment of MWCNTs inducement is highlighted through fortunate fracture from the joint position. The macrofibrous surface and micro localize examination confirms the pull-out mode of MWCNTs fracture that is further intimated in the transferring of the load from the matrix to fiber.
5. Different activated features were available at the fracture surfaces though all of them belong to the classical ductile fracture mode. Elliptical dimples completely in resemblance with cup and cone type behavior formed an isotropic flow of fractured surface almost in all specimens. However, microvoid coalescence is a predominant mode along with deep dimples and transgranular shear fractures with relatively shallow flattened dimples.
6. This study has provided an insight view of long aspect ratio MWCNTs inducement for the improvement of microstructure and fracto-mechanical behavior in the field of fusion welding. It has created a reference point for other upcoming studies and further results for carbonaceous and other related materials.

## Figures and Tables

**Table 1:** The chemical composition of Al-Mg-Si alloy in weight percentage.

**AA6061 (BM)**	**Si**	**Mg**	**Fe**	**Cu**	**Cr**	**Mn**	**Zn**
	0.6–0.8	1.0–1.2	≤ 0.4	≤ 0.2	≤ 0.2	≤ 0.2	≤ 0.1
**AA6061 (Tubes)**	**Si**	**Mg**	**Fe**	**Cu**	**Cr**	**Mn**	**Zn**
	1.0–1.2	0.9–1.0	≤ 0.3	≤ 0.2	≤ 0.2	≤ 0.2	≤ 0.1
